# Brain activations associated with fearful experience show common and distinct patterns between younger and older adults in the hippocampus and the amygdala

**DOI:** 10.1038/s41598-018-22805-9

**Published:** 2018-03-23

**Authors:** Chia-Shu Lin, Ching-Yi Wu, Shih-Yun Wu, Hsiao-Han Lin

**Affiliations:** 1 Department of Dentistry, School of Dentistry, National Yang-Ming University, Taipei, Taiwan; 2 Institute of Oral Biology, School of Dentistry, National Yang-Ming University, Taipei, Taiwan; 3 Division of Family Dentistry, Department of Stomatology, Taipei Veterans General Hospital, Taipei, Taiwan

## Abstract

Revisiting threat-related scenes elicits fear and activates a brain network related to cognitive-affective processing. Prior experience may contribute to the present fearful experience. We aimed to investigate (a) patterns of brain activation associated with individual differences in past fearful experiences (pFear) and the present fear elicited by watching videos (eFear) and (b) age-related differences in the activation patterns. Forty healthy adults, including 20 younger adults (YA) and 20 older adults (OA), underwent functional magnetic resonance imaging while watching videos containing high- and low-threat scenes of medical treatment. Both age subgroups showed positive correlations between pFear and bilateral hippocampal activation. Only YA showed threat-related activation in the bilateral anterior insula and activation positively correlated with pFear in the bilateral S1 and the amygdala. The evidence suggests that the hippocampus, amygdala and S1 may play key roles in bridging past fearful experiences and the present fear elicited by revisiting visual scenes and that the interaction between memory and emotional processing may be age dependent.

## Introduction

Previous neuroimaging studies have revealed that increased threat (e.g., watching high-threat scenes contrasted with low-threat scenes) is associated with an increased activation in the anterior insula (aINS) and the dorsal anterior cingulate cortex (dACC)^1–4^. Activation of these regions is associated with recalling past pain, imagining self-pain and empathizing with others’ pain^2,5,6^. Meta-analytical evidence has revealed that both aINS and dACC activation was associated with the anticipation of pain, a critical element of anxiety^7^. In patients with a specific phobia, the aINS and the amygdala showed consistent activation when they responded to phobic stimuli^8^. Finally, a recent meta-analysis revealed that emotional processing is associated with the functional connectivity between the amygdala, the parahippocampus, the aINS and the anterior cingulate cortex^9^. The cumulating evidence suggests that the aINS, the dACC and the amygdala play a major role in the processing of threat-related information.

Though the neural mechanisms regarding threat processing have been widely studied, until now, the association between aging and the mechanisms of emotional processing has not been fully elucidated. Previous studies have revealed that younger and older people may differ in their behavioral and brain processing of threat-related information^10^. Compared to younger individuals, older individuals react less to negative conditions (i.e., a positivity effect)^10,11^, including watching emotional facial expressions^12–14^, feeling empathy to pain^15^, and watching emotional scenes^16^. The neural mechanisms underlying the age-related bias in emotional processing have not been fully elucidated. On the one hand, the differences in emotional processing may reflect an age-related decline in attentional control, primarily mediated by the prefrontal cortex^14,17^. For example, an age-related decline in structural connectivity was found between the amygdala and the prefrontal cortex^17^. In patients with post-traumatic stress disorder, increased age was associated with increased amygdala volume^18^ and decreased amygdala-prefrontal functional connectivity^19^. These studies have convergently revealed that increased age is associated with structural or functional changes in the frontal, insular and limbic regions^14,17–19^. On the other hand, the positivity effect may reflect the selective modulation of the memory of threat-related information as a method of emotion regulation for older people^11^. For example, activation of the medial temporal lobe, including the hippocampus, the parahippocampus, and the amygdala, is associated with retrieving emotional autobiographical events^20^, watching fearful faces^12^, and processing emotional words^21^. The role of aging in the association between memory and emotional processing has remained unclear.

**Figure 1:**
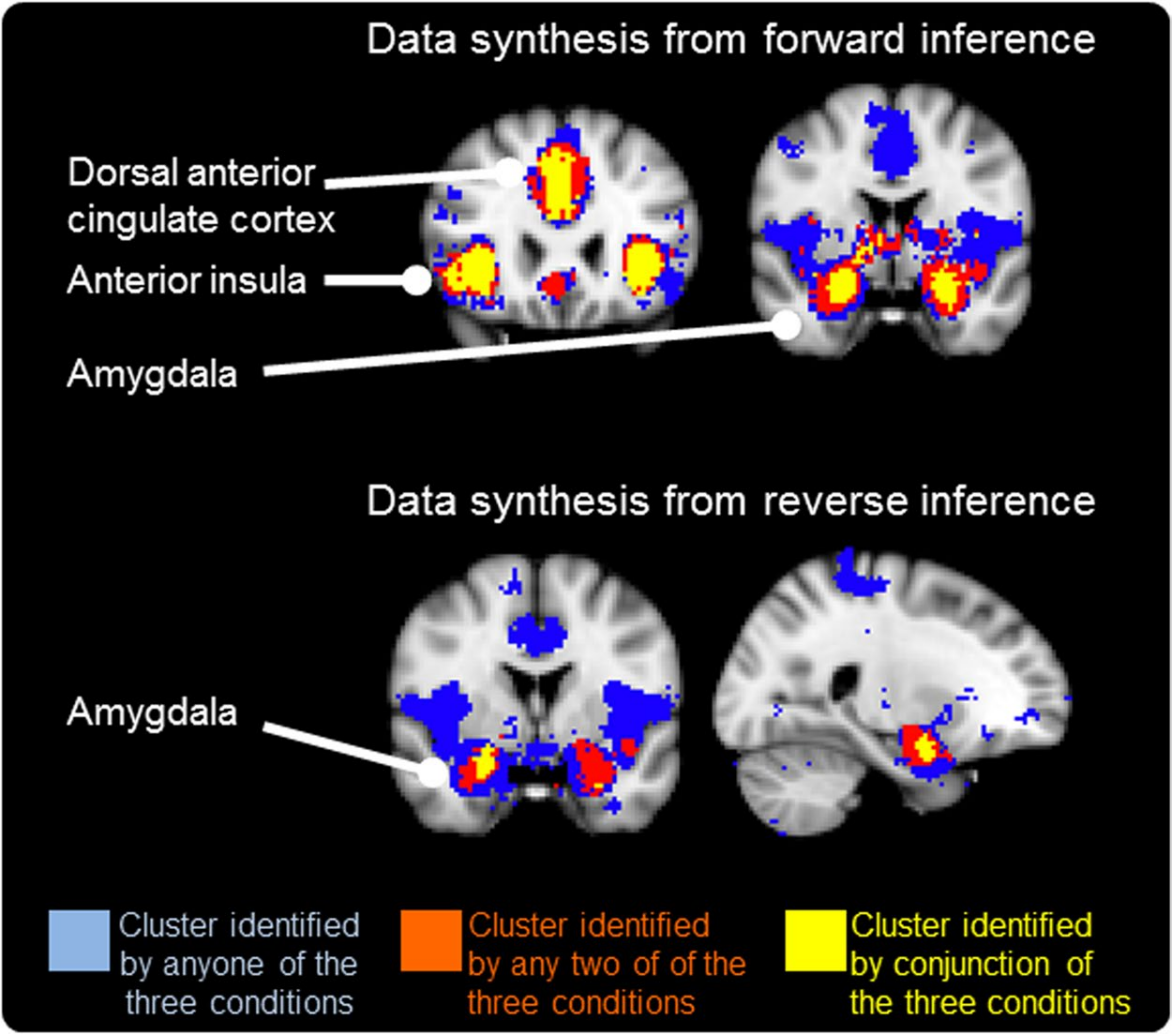
Selection of the regions of interest (ROIs). The pattern of brain activation was extracted from Neurosynth for the terms ‘fear’, ‘anxiety’ and ‘pain’, using a forward inference model (upper panel) and a reverse inference model (lower panel). The blue area denotes the activation corresponding to any of the three terms. The red area denotes the activation corresponding to any two of the terms. The yellow area denotes the activation common to all three terms. These regions included the bilateral anterior insula, the dorsal cingulate cortex, and the bilateral amygdala.

The current study focused on age-related differences in the processing of threat-related experiences. We hypothesized that a younger subgroup but not an older subgroup would show significant activation in the aINS, dACC and amygdala, which are commonly associated with the processing of threat-related information (Hypothesis I). The selection of the a priori region of interest (ROI) was based on the literature stated above, particularly the conclusions from imaging meta-analyses^2,7–9^, and the results of an automated large-scale meta-analysis, performed using the online platform Neurosynth (www.neurosynth.org) (see Methods and Fig. 1 for detailed information). Next, using a whole-brain exploratory analysis, we identified the brain activation patterns that were positively correlated with past fearful dental experience (pFear) and the current fear elicited by watching a video of dental treatment (eFear). We considered the activation corresponding to pFear to be the neural correlates of memories of past fearful experiences and the activation corresponding to eFear to be the neural correlates of fear elicited by current stimuli. Using an ROI-based approach, we tested the hypotheses that increased activation in the amygdala and hippocampus would be correlated with pFear in the younger but not older participants, signifying the selective modulation of threat-related memories (Hypothesis II). Finally, we hypothesized that increased activation in the aINS and dACC would be correlated with eFear in the younger but not older participants, signifying the positivity effect in emotional processing (Hypothesis III).

## Materials and Methods

### Participants

Forty healthy participants (27 females; min.–max. age = 23–74 years) were recruited for a functional magnetic resonance imaging (fMRI) scan. All participants were screened for the same exclusion criteria used in our previous studies^3^: (1) having a history of major physical or psychiatric disorders including epilepsy, major depression, schizophrenia or neurovascular diseases; (2) having a history of brain injury or having undergone brain surgery; and (3) being unable to undergo MRI because of physical (e.g., having a surgical implant) or psychological (e.g., claustrophobia) contraindications. The participants were recruited via advertisements posted at the university campus and local community centers. Before participating in the study, all participants provided written informed consent, which was approved by the Institutional Review Board of National Yang-Ming University (ID: YM102030E). All experiments were performed in accordance with relevant guidelines and regulations.

To investigate the age-related differences in emotional experiences and brain mechanisms, the participants were classified into two age subgroups by their median age (48.0): younger adults (YA, aged < 48.0 years) and older adults (OA, aged ≥ 48.0 years). Rather than adopting the participants’ age as a continuous variable, we separated them into two age subgroups because of the non-normal distribution of age (See Results and Table 1). A total of 20 participants were included in each of the OA and YA subgroups.

**Table 1:** Demographic and Behavioral Profiles of the Study Groups.

All (40)	F/M (27/13)	Age	pFear	pPain	eFear	FDV	MDAS
Mean		44.4	4.7	4.5	4.1	3.0	12.2
Median		48.0	4.7	4.7	3.8	2.5	12.0
Std		18.1	2.4	2.0	2.1	2.3	4.1
Min		23	0.0	0.0	−0.17	0	5
Max		74	9.7	8.3	7.8	10	22
Normality^a^		<0.001	0.797	0.122	0.302	0.004	0.120
**Older (20)**	**F/M (16/4)**						
Mean		61.0	4.0	4.1	3.6	2.8	10.7
Median		59.0	4.2	4.5	3.2	2.0	10.5
Std		7.3	2.6	2.2	2.5	2.4	3.6
Min		50	0.0	0.0	−0.2	0	5
Max		74	9.7	8.3	7.8	8	18
Normality^a^		0.112	0.454	0.069	0.276	0.020	0.464
**Younger (20)**	**F/M (11/9)**						
Mean		27.8	5.3	4.9	4.5	3.3	13.8
Median		27.0	5.7	4.7	4.3	3.0	12.0
Std		5.8	2.1	1.8	1.6	2.1	4.2
Min		23	1.0	1.7	2.7	0	8
Max		46	8.0	8.0	7.7	10	22
Normality^a^		0.001	0.190	0.422	0.048	0.008	0.175
Between sub-group comparison^b^							
	**Gender**						
p value	0.177	<0.001	0.094	0.224	0.172	0.254	0.016

eFear, fear elicited by the video paradigm; FDV, frequency of visiting a dentist in the past two years; MDAS, the score of the Modified Dental Anxiety Scale; PCS, the score of the Pain Catastrophizing Scale; pFear, fear of prior dental treatment; pPain, pain of prior dental treatment; Std, standard deviation.

^a^Normality was examined using the Shapiro-Wilk test.

^b^Between-group comparison was performed using the Chi-square test with Yates’ correction for Gender, the independent t-tests for pFear, pPain, eFear, MDAS, and the Mann-Whitney U test for age and FDV.

### Pre-scan behavioral assessment

Before scanning, we assessed the participants’ past fearful experiences related to dental procedures using a customized questionnaire. We focused on three common dental procedures: having a tooth drilled (for dental filling), receiving ultrasonic scaling (for dental cleaning), and receiving an injection (for local anesthesia), which are considered common and stressful procedures, as evaluated in the Modified Dental Anxiety Scale (MDAS)^22^. The participants were asked to recall (a) how fearful they were and (b) how painful the procedure was based on their past experience using a 0–10 numeric rating scale (0 = no fear/pain, 10 = extremely fearful/painful). The scores were indexed as pFear (fear from prior experience) and pPain (pain from prior experience). Here, pPain was assessed to validate the score of pFear, because pain is widely regarded to be the major source of fear related to dental treatments^23^. Subsequently, the participants completed the MDAS, an assessment of trait dental anxiety^24^ with good internal consistency (0.89) and test-retest reliability (0.82)^25^.

Before the fMRI scan, participants were interviewed about their personal experiences receiving dental treatment by the experimenter C-S Lin, a dentist of general practice. The number of dental visits during the past two years was used as an index of the frequency of dental visits (FDV). The interview ensured that all the participants understood the dental procedures that would be presented in the video scenes during the fMRI experiment.

### FMRI task and procedures

**Figure 2:**
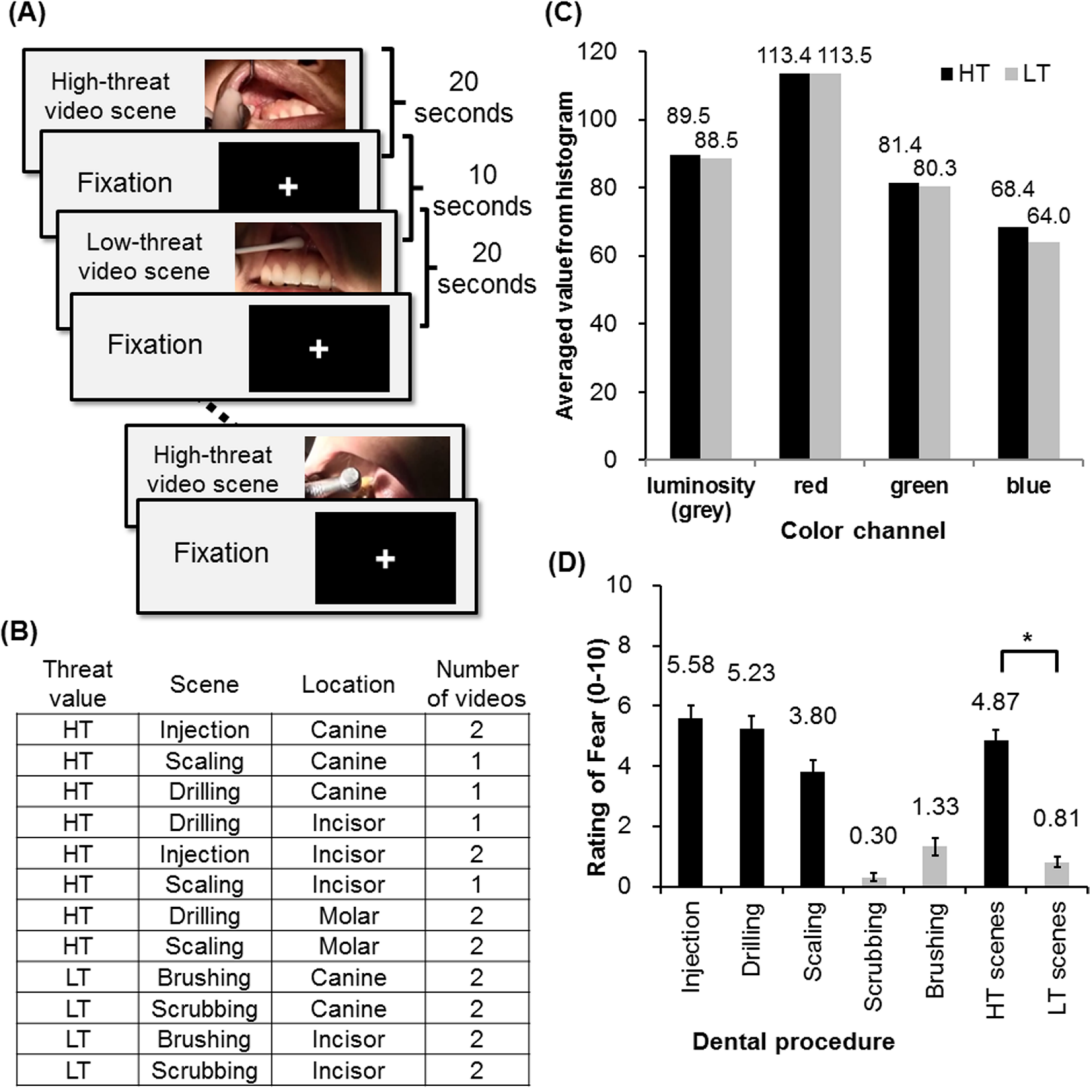
Experimental design. **(A**) The fMRI experiment adopted a block design consisting of 12 blocks of high-threat (HT) video scenes, 8 blocks of low-threat (LT) video scenes, and 20 blocks of fixation. **(B)** Video scenes of the threat-related blocks. (**C**) Assessment of the colorimetric properties revealed that the luminosity and colors of the HT and the LT scenes were well-balanced. (**D**) The ratings of fear elicited by watching the video. The HT scenes (including injection, drilling and scaling) elicited significantly stronger fear compared to the LT scenes. The asterisk denotes P value < 0.001 (two-tailed paired t-test).

We adopted a block design for presenting the video scenes (Fig. 2A). The visual stimuli were delivered using a mirror that reflected the screen from a projector. The auditory stimuli were delivered using earphones. The presentation of visual and auditory stimuli was synchronized using the software Presentation^®^ (Version 18.0, Neurobehavioral Systems, Inc., Berkeley, CA, www.neurobs.com). During the fMRI scan, the participants were instructed to watch a series of video scenes depicting dental procedures, and they were instructed to imagine the fear and pain related to the scenes as if they were receiving the procedure. The videos consisted of high-threat (HT) (e.g., receiving dental injection) and low-threat (LT) (e.g., brushing teeth) scenes, each lasting for 20 seconds. All the five procedures are common dental procedures that most of the participants would experience in the past. Three of them are deemed as HT procedures and the other two as LT procedures, based on the consensus of the dentists/co-authors (C-S Lin, C-Y Wu and S-Y Wu). Four video scenes were created for each procedure. The whole scan consisted of 12 HT blocks, 8 LT blocks, and 20 fixation blocks, in which a fixation cross was presented for 10 seconds. Each HT or LT block was followed by a fixation block. The order of the video blocks was pseudo-randomly organized and assigned consistently to all participants. The content of the HT and LT video scenes is listed in Fig. 2B. The video scenes from the HT and the LT subgroups were balanced for luminosity and the histogram of the primary colors (red, green and blue). The average values of luminosity and the primary colors, extracted from the initial frame of each scene, were assessed using the freeware InfraView Version 4.40 (http://www.irfanview.com). Assessment of the colorimetric properties revealed that the luminosity and colors of the HT and the LT scenes were well balanced (Fig. 2C).

### Post-scan behavioral assessment

After scanning, we assessed the participant fear elicited by watching the video scenes using the same rating scale adopted in the pre-scan behavioral assessment. The participants watched the snapshots from the video scenes that they have seen during fMRI scan and rated how fearful they experienced. The videos scenes that depicted injection, drilling and scaling elicited a significantly higher fear score compared to the scenes that depicted brushing and scrubbing (Fig. 2D). Therefore, the first three scenes were combined as the HT blocks, and the latter two scenes were combined as the LT blocks. A paired t-test showed that fear was significantly higher for the HT blocks compared to the LT blocks (two-tailed P < 0.001, Fig. 2D). To each participant, we defined the index of elicited fear (eFear) as the self-reported fear aroused from the HT procedures (eFear_HT_, including injection, drilling and scaling), compared to the self-reported fear aroused from the LT procedures (eFear_LT_, including brushing and scrubbing):

$${\rm{eFear}}={{\rm{eFear}}}_{{\rm{HT}}}-{{\rm{eFear}}}_{{\rm{LT}}}$$

therefore, both pFear and eFear represented the fear aroused from the HT procedures (i.e., injection, drilling and scaling).

### Acquisition and pre-preprocessing of imaging data

The imaging data were acquired on a 3-Tesla imaging system (Tim Trio, Siemens, Erlangen, Germany) with a quadrature head coil using the same parameters as reported in our previous study^3^. In general, functional data were acquired with T2-weighted gradient-echo EPI using blood-oxygenation-level-dependent contrast (TR/TE/flip angle = 2000 ms/20 ms/90°, matrix size = 64 × 64 × 40, voxel size = 3.4 × 3.4 × 3.4 mm^3^). Anatomical data were acquired with T1-weighted 3D gradient-echo pulse sequence (modified driven equilibrium Fourier transform: TR/TE/TI = 2530/3.03/1100 ms, matrix size = 256 × 256 × 192, voxel size = 1 × 1 × 1 mm^3^).

Functional imaging data were pre-processed and analyzed using Statistical Parametric Mapping (SPM8, the Wellcome Trust Centre for Neuroimaging, London, http://www.fil.ion.ucl.ac.uk/spm), based on the same published protocol ^3^. Scans were slice-time corrected, realigned and co-registered to the individual T1-weighted anatomical image before being normalized to a 2 × 2 × 2 mm Montreal Neurological Institute (MNI) space. Scans were further smoothed using a Gaussian kernel (full-width-at-half-maximum = 8 × 8 × 8 mm), high-pass filtered, and corrected for temporal serial correlations.

### Analysis of behavioral data

For all the scores from the behavioral assessments, we performed the Shapiro-Wilk test to investigate the normality of the score distribution. The score was considered non-normally distributed if the null hypothesis was rejected, with an alpha level = 0.1. To investigate the strength of the association between the behavioral scores, we used Pearson’s correlation coefficient r for bivariate-normally distributed scores or the Spearman’s correlation coefficient rho for non-normally distributed scores. Comparisons between the age subgroups were performed using independent t-tests (for normally distributed scores) or Mann–Whitney U tests (for non-normally distributed scores).

### Determination of the regions of interest

The ROI-based analysis focused on the brain activation of the aINS, dACC and amygdala. The selection was partly based on the results of automated large-scale meta-analysis, performed using Neurosynth. We first looked for experiments associated with the following mental constructs (i.e., ‘term’): ‘fear’, ‘anxiety’, and ‘pain’. The meta-analytical results were retrieved for both the ‘forward inference’ model and the ‘reverse inference’ model. The forward inference model provided the pattern of brain activation consistently found when the mental construct was associated. The reverse inference model provided the brain activation pattern that represents the likelihood that the mental construct was associated, given the presence of reported activation^26^. The brain activation maps were binarized, and a conjunction was made between the three constructs (i.e., anxiety ∩ fear ∩ pain) for the forward and the reverse inference models. As shown in Fig. 1, we found that in the forward inference model, the bilateral aINS, the bilateral dACC and the bilateral amygdala were consistently activated across the three constructs. The right amygdala was predictive of the three mental constructs (Fig. 1). Therefore, the current study adopted the aINS, the dACC and the amygdala for testing our ROI-specific hypothesis.

### Analysis of the fMRI data

For all the participants, we modeled a first-level general linear model (GLM) that comprised (a) 8 blocks of watching HT video scenes (20 seconds per block), (b) 8 blocks of watching LT video scenes (20 seconds per block), (c) 16 fixation blocks (10 seconds per block), and (d) the motion parameters (three for rotations and three for translations) obtained from image realignments as nuisance regressors. To balance the number of HT vs. LT blocks, we excluded 4 blocks of the ‘scaling’ scenes from the HT blocks, which showed moderate fear ratings (Fig. 2D). To examine the effect of the imbalance of the number of HT vs. LT blocks, we performed an additional analysis by including the 12 HT blocks (i.e., including the 4 blocks of the scaling scenes). The results of the additional analysis (i.e., 12 HT blocks vs. 8 LT blocks) were reported in Supplementary Table S1 and Table S2.

We performed the following three analyses:

1. ROI-based analysis of brain activation of the threat-related effect: To test Hypothesis I, the contrasting images were assessed by a one-sample t-test, independently for the YA and the OA subgroups, with gender and age as nuisance regressors. The result was initially thresholded by intensity (P_uncorrected_ < 0.001). A small volume correction was applied, separately, to the bilateral aINS dACC, and amygdala ROIs. The activation was considered statistically significant at P_FWE-corrected_ (corrected for familywise error) < 0.05. The ROIs were manually defined in an independent study sample, based on our previous studies^27^.
2. Whole-brain exploratory analysis of brain activation associated with pFear/eFear: We focused on the association between pFear and eFear and the threat-related contrasting images (i.e., HT vs. LT). The pFear/eFear scores and the participants’ gender and age (i.e., the nuisance regressors) were modeled as the covariates. For the whole-brain explorations, we considered a cluster to be statistically significant by an initial intensity threshold of P_uncorrected_ < 0.001 and a corrected size threshold P_FWE-corrected_ < 0.05, based on published recommendations^28^. Both positive correlation and negative correlation results were investigated using the same threshold. The whole-brain exploratory analyses were performed for all participants (N = 40) and separately for the OA (N = 20) and YA (N = 20) subgroups.
3. ROI-based analysis of age-related patterns of activation: Based on the results from Analysis 2 (see Results), we performed ROI analyses on the bilateral hippocampus, the bilateral primary somatosensory cortex (S1), and the bilateral amygdala. It should be noted that the ROIs were defined according to the Jülich histological atlas (for S1) and the Harvard-Oxford subcortical structural atlas (for the hippocampus) from FslView (https://fsl.fmrib.ox.ac.uk/fsl/fslwiki/FslView), rather than the cluster found in the whole-brain analyses. The use of an independent source of ROIs avoids the non-independence in ROI analysis^29^.
  For each age subgroup and each ROI, we extracted the mean brain activation averaged from all the voxels within the corresponding mask using the toolkit REX (https://www.nitrc.org/projects/rex). Analyses of Pearson’s correlation were performed between the scores of pFear (Hypothesis II) and eFear (Hypothesis III) and the mean activation from each of the six ROIs, separately for the OA and the YA subgroups.

## Results

### Behavioral results

The demographic and behavioral profiles of the participants are shown in Table 1. We found no statistically significant differences between age subgroups in the participants’ gender (chi-square test, p = 0.177) or in the scores of pFear, pPain and eFear (Table 1). The OA subgroup showed a significantly higher age (median = 59 years), compared to the YA subgroup (median = 27 years) (Mann-Whitney U test p < 0.001, η2 = 0.75). The OA subgroup showed lower scores of trait dental anxiety (mean MDAS score ± standard deviation = 10.7 ± 3.6) compared to the YA subgroup (13.8 ± 4.2) (two-tailed independent t-test p < 0.05, Cohen’s d = −0.79).

**Table 2:** Results of whole-brain exploratory analyses.

Cluster		Peak intensity				Region
Size (voxel)	P_FWE_	Z score	x	y	z	
**(A) Brain activation of threat-related experience**						
High-threat > Low-threat scenes, OA (N = 20)						
945	0.000	4.8	30	−88	−10	Visual Cortex
		4.1	18	−92	−16	Visual Cortex
		4.0	22	−84	−16	Visual Cortex
485	0.004	4.7	−10	−70	54	Visual Cortex
		3.8	2	−58	50	Precuneus
		3.5	−6	−60	48	Precuneus
1283	0.000	4.3	−48	−68	−22	Visual Cortex
		4.2	−22	−98	−14	Visual Cortex
		4.1	−32	−92	−14	Visual Cortex
Low-threat > High-threat scenes, OA: n.s.						
High-threat > Low-threat scenes, YA (N = 20)						
345	0.042	4.9	28	−94	−6	Visual Cortex
1170	0.000	4.0	30	22	0	Insula
		4.0	20	2	12	Putamen
		3.9	40	24	12	Inferior Frontal Gyrus
Low-threat > High-threat scenes, YA: n.s.						
**(B) Brain activation associated with pFear**						
Positive correlation with pFear, all participants (N = 40)						
589	0.007	4.5	−26	−6	−24	Hippocampus
		4.4	−24	−12	−14	Hippocampus
		4.1	−32	−18	−16	Hippocampus
443	0.022	4.0	24	−12	−20	Hippocampus
		3.9	26	−6	−26	Hippocampus
		3.6	42	−8	−22	Parahippocampus
Negative correlation with pFear, all participants: n.s.						
Positive correlation with pFear, OA: n.s.						
Negative correlation with pFear, OA: n.s.						
Positive correlation with pFear, YA: n.s.						
Negative correlation with pFear, YA: n.s.						
**(C) Brain activation associated with eFear**						
Positive correlation with eFear, all participants (N = 40)						
1182	0.000	4.6	−32	−30	50	S1
		3.9	−30	−50	70	Superior parietal lobe
		3.6	−46	−30	56	S1
Negative correlation with pFear, all participants: n.s.						
Positive correlation with eFear, OA (N = 20)						
521	0.002	4.3	−32	−48	74	Superior parietal lobe
		4.1	−32	−30	52	S1
		3.7	−22	−46	66	Superior parietal lobe
Negative correlation with eFear, OA: n.s.						
Positive correlation with eFear, YA: n.s.						
Negative correlation with eFear, YA: n.s.						

S1, primary somatosensory cortex.

**Figure 3:**
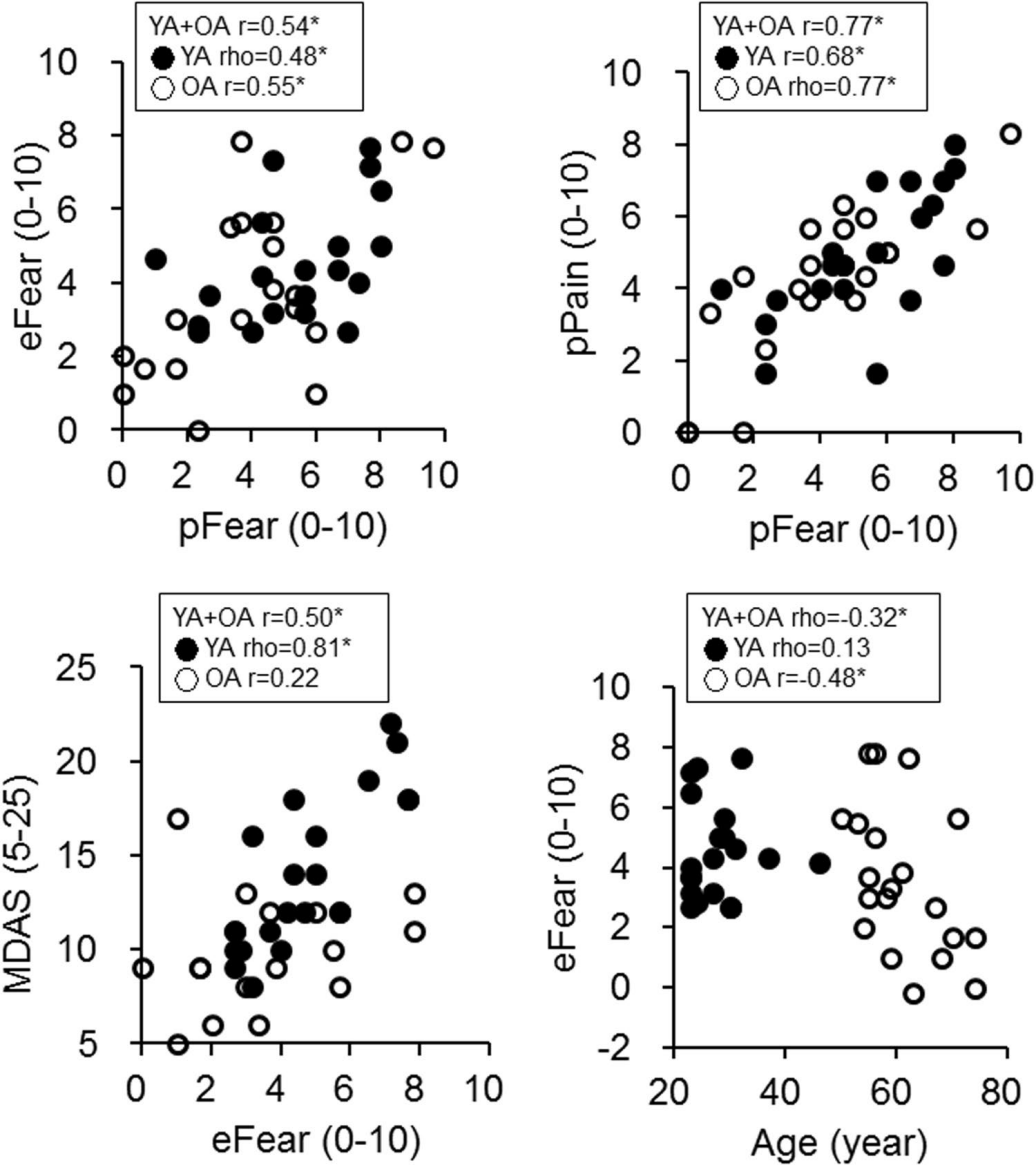
Behavioral results. There were significant correlations between eFear and pFear and between pFear and pPain in both the younger adults (YA, solid circles) and the older adults (OA, open circles). In the YA subgroup, the MDAS score was positively correlated with eFear. In the OA subgroup, age was negatively correlated with eFear.

In terms of the association between behavioral scores, pFear was positively correlated with eFear (r = 0.54, p < 0.001). Additionally, pFear was positively correlated with pPain (r = 0.77, p < 0.001) (Fig. 3A). The findings suggested that the fear elicited by watching video scenes was associated with one’s prior experience of the treatment. Additionally, the MDAS score was positively correlated with pFear (r = 0.61, p < 0.001). Notably, we found an age-related difference in the behavioral patterns: (a) eFear was negatively correlated with age in the OA subgroup (r = −0.48, p = 0.034) but not in the YA subgroup (rho = 0.13, p = 0.59) (Fig. 3B), and (b) the MDAS score and eFear were positively correlated in the YA subgroup (rho = 0.81, p < 0.001) but not in the OA subgroup (r = 0.22, p = 0.34) (Fig. 3B).

### Imaging results

#### Analysis 1: ROI-based analysis of the threat-related effect

In the YA subgroup, we found significant activation in the left ([x,y,z] = [−28 24 4], z = 3.99, P_FWE-corrected_ = 0.002, corrected for small volume) and the right aINS ([x,y,z] = [30 22 0], z = 4.04, P_FWE-corrected_ = 0.002, corrected for small volume). No significant activation was found in the bilateral dACC or amygdala. An additional whole-brain exploratory analysis revealed threat-related activation in the visual cortex, the putamen, the inferior frontal gyrus (Table 2). In the OA subgroup, no significant activation was found in the ROIs. The whole-brain exploratory analysis revealed threat-related activation in the visual cortex and the precuneus (Table 2).

#### Analysis 2-1: Brain activation associated with pFear

**Figure 4:**
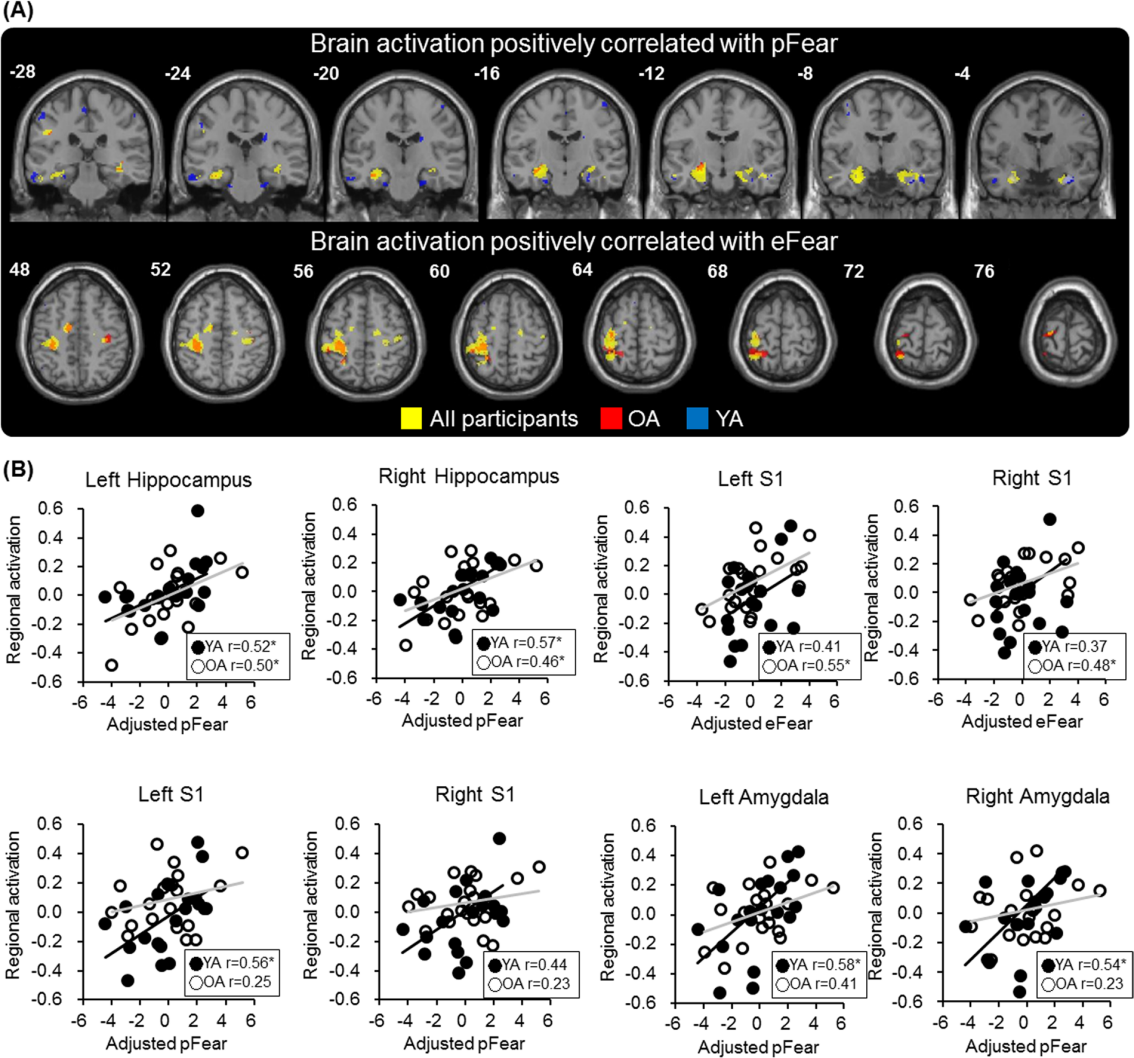
Imaging results. (**A**) Whole-brain exploratory analyses revealed that pFear was positively correlated with threat-related hippocampal activation. Note that the finding was consistent for both the OA (yellow) and the YA (green) subgroups. In contrast, eFear was positively correlated with activation in the primary somatosensory cortex only for the YA subgroup. **(B**) The ROI-based analyses revealed a consistent pattern. The association between pFear and hippocampus/amygdala activation showed an age-related difference.

For all participants, the whole-brain exploratory analysis revealed that pFear was positively correlated with activation in the left amygdala, extending to the hippocampus ([x,y,z] = [−26, −6, −24], z = 4.5, cluster-level p_FWE-corrected_ = 0.007, size = 589 voxels) and the right hippocampus ([x,y,z] = [24, −12, −20], z = 4.0, cluster-level p_FWE-corrected_ = 0.022, size = 443 voxels). No above-threshold clusters were found for the negative contrast (Table 2 and Fig. 4A).

#### Analysis 2-2: Brain activation associated with eFear

For all participants, the whole-brain exploratory analysis revealed that eFear was positively correlated with activation in the left S1 ([x,y,z] = [−32, −30, 50], z = 4.6, cluster-level p_FWE-corrected_ < 0.001, size = 1182 voxels) but not the right S1. No above-threshold clusters were found for the negative contrast (Table 2 and Fig. 4A).

#### Analysis 3: ROI-based analysis of age-related differences

Based on the findings from the whole-brain exploratory analyses described above (Table 2), we focused on the brain activation of the bilateral hippocampus, the S1 and the amygdala. Brain activation in the bilateral hippocampus was positively correlated with pFear for the YA subgroup (left: r = 0.52, p = 0.019, coefficient of determination = 0.27; right: r = 0.57, p = 0.009, coefficient of determination = 0.32; pFear adjusted for age and gender effect) and the OA subgroup (left: r = 0.50, p = 0.023, coefficient of determination = 0.25; right: r = 0.46, p = 0.041, coefficient of determination = 0.21) (Fig. 4B). The findings generally concurred with the results from the additional whole-brain exploratory analyses, which showed pFear-related clusters in the hippocampus and the amygdala in the YA subgroup (Table 2).

In terms of age-related differences, in the YA subgroup, pFear was positively correlated with left S1 activation (r = 0.56, p = 0.011, coefficient of determination = 0.31) and the association between pFear and right S1 showed a trend of significance (r = 0.44, p = 0.051, coefficient of determination = 0.19). In the OA subgroup, the correlations were not statistically significant (r = 0.25, p = 0.29) (Fig. 4B). pFear was positively correlated with bilateral amygdala activation only in the YA subgroup (left: r = 0.58, p = 0.007, coefficient of determination = 0.34; right: r = 0.54, p = 0.014, coefficient of determination = 0.29). In the OA subgroup, the correlations were not statistically significant (left: r = 0.41, p = 0.07; left: r = 0.23, p = 0.32) (Fig. 4B). Finally, eFear was positively correlated with the bilateral S1 activation only in the OA subgroup (left: r = 0.55, p = 0.011, coefficient of determination = 0.31; right: r = 0.48, p = 0.034, coefficient of determination = 0.23; eFear adjusted for age and gender effect). In the YA subgroup, the correlations were not statistically significant (left: r = 0.41, p = 0.08; right: r = 0.37, p = 0.11) (Fig. 4B).

## Discussion

### Summary of the major findings

1. Common to both age subgroups, when threat-related experience was elicited by watching the scenes, bilateral hippocampal activation was positively correlated with pFear.
2. In terms of age-related differences, the YA subgroup showed a significant threat-related activation in the bilateral aINS, consistent with previous findings about the processing of threat-related experiences. Brain activation in the bilateral S1 and amygdala was positively correlated with pFear.
3. In contrast, the OA subgroup did not show a significant threat-related activation in the bilateral aINS. Brain activation in the amygdala was not significantly correlated with pFear. In contrast, brain activation in the bilateral S1 was significantly correlated with eFear.

In general, the current evidence revealed that, behaviorally and neuro-scientifically, prior fearful experience was associated with the present fear elicited by revisiting the medical scenes. Moreover, age may play a key role in the interaction between the past and present experiences.

### The hippocampus and the amygdala: bridging the past and the present

The current study focused on the threat-related experience associated with medical treatment. We used scenes of dental procedures to elicit fear. It is widely known that dental fear is associated with past traumatic experiences of dental treatment^30^. We found that higher pFear was associated with stronger activation in the bilateral hippocampus and amygdala, which have been considered to be core components of emotion and memory processing^31^. In line with our findings, when pain-related threat was acquired via fear conditioning (i.e., a situation where one has actually experienced pain), the threat-related hyperalgesic effect was associated with activation in the hippocampus and the amygdala, compared to a threat acquired via verbal instruction (i.e., a situation where one has not experienced pain)^32^. Bilateral amygdala activation during the anticipation of pain is associated with the personal trait harm avoidance, and hippocampal activation is associated with individual sensitivity to pain expectancy^33^. Consistently, dental procedures are commonly considered frightening and invasive to many patients^30,34^, and excessive fear and worry could lead to an avoidance of treatment^35^. Our results suggest that activation in these regions may reflect individual differences in retrieving past threat-related experiences.

Notably, the hippocampus showed a functional segregation along its longitudinal axis. While its dorsal-posterior part is crucial to processing spatial memory, its ventral-anterior part plays a key role in mediating anxiety-related behaviors^36^. Concordantly, pFear-related activation was found bilaterally in the anterior part of the hippocampus (Table 2 and Fig. 4A). We noticed that amygdala activation was only associated with pFear in the YA but not the OA subgroup (Fig. 4B). The coactivation of the hippocampus and the amygdala suggested that in the YA subgroup, the activity of the limbic system may signify a stronger integration of the context and threat value.

### Age-related difference in the S1 activation

S1 activation has been widely considered to be the major cortical representation of pain when one receives nociceptive stimuli^37^. However, recent evidence suggests that S1 activation could be associated with a painful experience without a direct nociceptive input, as elicited by recalling past pain^5^ or by empathizing with pain-related pictures^2^. Moreover, increased S1 activation has been associated with unpredictable pain compared to predictable pain^38^, and the unpredictability was associated with increased anxiety toward pain^39^. S1 activation also reflects individual differences in the sensitivity of pain expectancy^33^. The findings suggest that S1 activation may signify the threat-related experience associated with pain, rather than the nociceptive input per se. In line with this view, our findings showed that for the OA subgroup, individual differences in S1 activation would reflect the perceived threat (eFear) related to invasive medical procedures. For the YA subgroup, bilateral S1 activation was correlated with pFear, but not eFear. A potential explanation is that for the YA subgroup, individual differences in trait dental anxiety would contribute to their perceived threat, evidenced by the strong association between the MDAS score and eFear (Fig. 3). We also noticed that the YA subgroup showed a higher MDAS score, compared to the OA subgroup (Table 1). Therefore, when watching the video scenes, the YA subgroup may perceive a stronger threat-related experience not only to the HT scenes but also to the LT scenes, due to the averagely higher level of anxiety.

Notably, we found a significant association between pFear and the bilateral amygdala as well as S1, only for the YA subgroup (Fig. 4B). The connection between the amygdala and S1 plays a key role in the limbic system, signifying the integration of pain (as a primary reinforcer) in emotional processing^31^. The age-related difference may suggest that in the younger participants, the amygdala may play a key role in bridging past experiences (pFear) with a currently encountered threat (eFear). In contrast, in the OA subgroup, pFear was not significantly correlated with either the S1 or the amygdala. The findings imply that, in terms of processing of threat-related memories, older participants may differ from younger participants both in the processing of sensory information and in the processing of emotional information.

### Aging and the effect of affective positivity

Behavioral and neuroimaging evidence has revealed an age-related ‘positivity effect’ on emotional processing. Compared with younger adults, older adults tend to pay more attention to positive information, and such a preference may be associated with attention processing and emotion regulation^10,40^. Our results partially concurred with these findings. We found that the age subgroups, even though they did not show a significant difference in the pFear and eFear behavioral scores (Table 1), revealed a different pattern of brain activation corresponding to pFear and eFear. S1 and amygdala activation were positively correlated with pFear in the YA subgroup but not the OA subgroup (Table 2 and Fig. 4). Such a ‘blunt’ response in the S1 and the amygdala may suggest that the bottom-up processing of pain and fear is less sensitive in the older participants.

In contrast, our imaging results did not show any above-threshold clusters in the contrasting images that negatively correlated with either pFear or eFear. We also did not find threat-related activation in the medial prefrontal cortex (MPFC), a region critically associated with age-related emotional regulation^21,40^. A potential explanation for the lack of MPFC activation is that the dental procedures presented in our videos related to a long-term benefit in oral healthcare. Emotionally and motivationally, the older participants may spontaneously adapt themselves to the situation, without further effort to regulate their emotions. Behaviorally, compared to the YA subgroup, the OA subgroup did not differ in the frequency of recent dental visits (Table 1). This behavioral pattern implied that, through their lifetime, older participants may have learned to efficiently cope with dental treatment, and therefore less additional regulatory effort is needed.

### Clinical implications

Watching a video that depicts negative scenes (e.g., a finger being cut) elicits feelings of pain and fear and an extensive brain network related to cognitive-affective processing^2^. Likewise, in a medical situation, patients develop strong fears when they revisit the same treatment procedures that had previously been painful and unpleasant (e.g., dental procedures). The patients may recall their past negative experiences and relate them to the present situation^30^. Excessive fear and worry could eventually lead to the avoidance of further treatment and deterioration of health^35^. In neuroimaging research, the video-provocation paradigm, including visual and auditory stimuli of scenes of dental treatment, was used to elicit increased fear and brain activation in an extensive network involved in pain and fear processing^1,3,4^. Most of the current studies elicited pain and fear using standardized pictures that depict scenes of injury or illness^2^. These scenes are associated with daily life and therefore elicit a common emotional experience. In contrast, in medical situations, the threat value of a procedure is highly subjective based on one’s prior experience. For example, previous exposure to pain has been associated with empathy to pain^41^, and prior experience of dental treatment has been associated with fear and avoidance toward dental scenes^3^. The brain mechanisms that bridge the past (prior experience of fear) with the present (the elicited fear) experiences remain unknown. Furthermore, behavioral and neuroimaging evidence has revealed that age may play a key role in modulating emotional experiences. It remains unknown how the age factor influences the interaction between one’s prior experience of fear and the presently elicited fear.

### Limitations and further considerations

First, our participants were recruited from the university campus and local community centers, rather than dental clinics. Therefore, the results may not fully capture patients with a high degree of dental fear. Judging from the normality of distribution of the MDAS score (Table 1), we consider our study sample to be representative of a community-based population, in which most people show only mild to moderate degrees of dental anxiety.

Second, the current results should be carefully interpreted due to the ecological validity of the study design. Similar to previous studies (e.g.^1,2^), we selected dental procedures that were commonly experienced by most of the participants. We deliberately excluded some less common but affectively stronger procedures (e.g., surgical extraction of a tooth). Disease-related scenes that may induce emotional experiences other than fear (e.g., disgust) were also excluded. Therefore, the findings should be interpreted based on the limitations in the material preparation.

Finally, we did not investigate the strategies of re-appraisal or emotional regulation that the participants adopted when they were watching the videos. Therefore, it is difficult to elaborate on how the older participants maintain an age-related affective positivity from the current results. The issues regarding individual strategies of re-appraisal or regulation are crucial in emotion processing in the medical context, because in a realistic scenario, patients need to balance both pain (i.e., fear of a procedure) and gain (i.e., treatment effect) at the same time. Such a complex interaction would require future investigations.

## Conclusion

The behavioral and imaging evidence suggests that present fear elicited by revisiting medical scenes was associated with past fearful experiences, and the hippocampus, amygdala and S1 may play a key role in bridging the past and the present fearful experience. Such an interaction between memory and emotional processing may be age dependent.

## Electronic supplementary material

Supplementary Information
