# Supplementary material for: Brain activations associated with fearful experience show common and distinct patterns between younger and older adults in the hippocampus and the amygdala
Source: Sci Rep. 2018 Mar 23;8:5137. doi: 10.1038/s41598-018-22805-9 (PMC5865205; doi:10.1038/s41598-018-22805-9)
Supplement: Supplementary file 1 — Supplementary Information [file 41598_2018_22805_MOESM1_ESM.docx]

**Supplementary Information**

Brain activations associated with fearful experience show common and distinct patterns between younger and older adults in the hippocampus and the amygdala

**Authors**

Chia-Shu Lin (corresponding author), Ching-Yi Wu, Shih-Yun Wu, Hsiao-Han Lin

**Supplementary Table S1** Results of whole-brain exploratory analyses (12 High-threat blocks vs. 8 Low-threat blocks)

| 1. Brain activation of threat-related experience | | | | | | |
| --- | --- | --- | --- | --- | --- | --- |
| High-threat > Low-threat scenes, OA (N=20) | | | | | | |
| Cluster | | Peak intensity | | | | Region |
| Size (voxel) | P_FWE_ | Z score | x | y | z |  |
| 494 | 0.006 | 4.7 | 30 | -90 | -10 | Visual Cortex |
| 390 | 0.017 | 4.3 | -32 | -90 | -14 | Visual Cortex |
|  |  | 3.8 | -22 | -98 | -14 | Visual Cortex |
| Low-threat > High-threat scenes, OA: n.s. | | | | | | |
| High-threat > Low-threat scenes, YA (N=20) | | | | | | |
| Cluster | | Peak intensity | | | | Region |
| Size (voxel) | P_FWE_ | Z score | x | y | z |  |
| 389 | 0.020 | 4.7 | 28 | -94 | -4 | Visual Cortex |
| 1045 | 0.000 | 4.8 | -10 | 6 | 20 | Caudate |
|  |  | 3.9 | -24 | 8 | 2 | Putamen |
|  |  | 3.7 | -14 | -14 | 20 | Thalamus |
| 2028 | 0.000 | 4.6 | 40 | 24 | 12 | Inferior Frontal Gyrus |
|  |  | 4.4 | 20 | 0 | 16 | Putamen |
|  |  | 4.3 | 12 | 0 | 20 | Caudate |
| 605 | 0.003 | 4.1 | -46 | 4 | 24 | M1 / S1 |
|  |  | 3.9 | -48 | 24 | 28 | Inferior Frontal Gyrus |
|  |  | 3.8 | -54 | 6 | 30 | M1 / Premotor Cortex |
| 470 | 0.009 | 4.7 | -26 | -98 | -4 | Visual Cortex |
|  |  | 3.7 | -38 | -74 | 20 | Visual Cortex |
|  |  | 3.5 | -40 | -84 | 30 | Visual Cortex |
| Low-threat > High-threat scenes, YA: n.s. | | | | | | |
| (B) Brain activation associated with pFear | | | | | | |
| Positive correlation with pFear, all participants (N=40) | | | | | | |
| Cluster | | Peak intensity | | | | Region |
| Size (voxel) | P_FWE_ | Z score | x | y | z |  |
| 472 | 0.015 | 4.4 | -26 | -10 | -16 | Hippocampus |
|  |  | 4.0 | -30 | -26 | -16 | Hippocampus |
| 370 | 0.037 | 4.0 | 22 | -12 | -20 | Hippocampus |
|  |  | 4.0 | 36 | -6 | -22 | Hippocampus |
|  |  | 3.3 | 32 | 4 | -28 | Amygdala |
| Negative correlation with pFear, all participants: n.s. | | | | | | |
| Positive correlation with pFear, OA: n.s. | | | | | | |
| Negative correlation with pFear, OA: n.s. | | | | | | |
| Positive correlation with pFear, YA (N=20) | | | | | | |
| Cluster | | Peak intensity | | | | Region |
| Size (voxel) | P_FWE_ | Z score | x | y | z |  |
| 379 | 0.012 | 4.4 | 36 | -4 | -24 | Amygdala / Hippocampus |
|  |  | 3.8 | 24 | -22 | -26 | Hippocampus |
|  |  | 3.6 | 30 | -36 | -32 | Cerebellum V / VI |
| 333 | 0.021 | 4.1 | 16 | 46 | 24 | Frontal Pole |
|  |  | 3.5 | 12 | 36 | 26 | Medial Prefrontal Cortex |
|  |  | 3.3 | 34 | 34 | 36 | Middle Frontal Gyrus |
| Negative correlation with pFear, YA: n.s. | | | | | | |
| (C) Brain activation associated with eFear | | | | | | |
| Positive correlation with eFear, all participants (N=40) | | | | | | |
| Cluster | | Peak intensity | | | | Region |
| Size (voxel) | P_FWE_ | Z score | x | y | z |  |
| 393 | 0.030 | 4.3 | -32 | -30 | 50 | S1 |
|  |  | 3.5 | -34 | -18 | 52 | M1/S1 |
|  |  | 3.2 | -38 | -38 | 62 | S1 |
| Negative correlation with pFear, all participants: n.s. | | | | | | |
| Positive correlation with eFear, OA: n.s. | | | | | | |
| Negative correlation with eFear, OA: n.s. | | | | | | |
| Positive correlation with eFear, YA: n.s. | | | | | | |
| Negative correlation with eFear, YA: n.s. | | | | | | |

M1, primary motor cortex; S1, primary somatosensory cortex.

**Supplementary Table S2** Comparison of the results of region-of-interest analyses

1. 8 high-threat blocks (excluding the ‘scaling’ scenes) vs. 8 low-threat blocks

| YA | Hippocampus | | Amygdala | | S1 | |
| --- | --- | --- | --- | --- | --- | --- |
|  | Left | Right | Left | Right | Left | Right |
| pFear (r) | 0.52 | 0.57 | 0.58 | 0.54 | 0.56 | 0.44 |
| p-value | 0.02 | 0.01 | 0.01 | 0.01 | 0.01 | 0.05 |
| eFear (r) | 0.38 | 0.35 | 0.33 | 0.25 | 0.41 | 0.37 |
| p-value | 0.10 | 0.13 | 0.15 | 0.30 | 0.08 | 0.11 |
| OA | Hippocampus | | Amygdala | | S1 | |
|  | Left | Right | Left | Right | Left | Right |
| pFear (r) | 0.50 | 0.46 | 0.41 | 0.23 | 0.25 | 0.23 |
| p-value | 0.02 | 0.04 | 0.07 | 0.32 | 0.29 | 0.33 |
| eFear (r) | 0.28 | 0.27 | 0.32 | 0.38 | 0.55 | 0.48 |
| p-value | 0.24 | 0.25 | 0.17 | 0.10 | 0.01 | 0.03 |

(B) 12 high-threat blocks (including the ‘scaling’ scenes) vs. 8 low-threat blocks

| YA | Hippocampus | | Amygdala | | S1 | |
| --- | --- | --- | --- | --- | --- | --- |
|  | Left | Right | Left | Right | Left | Right |
| pFear (r) | 0.51 | 0.56 | 0.57 | 0.56 | 0.57 | 0.46 |
| p-value | 0.02 | 0.01 | 0.01 | 0.01 | 0.01 | 0.04 |
| eFear (r) | 0.34 | 0.33 | 0.30 | 0.23 | 0.40 | 0.34 |
| p-value | 0.15 | 0.15 | 0.20 | 0.32 | 0.08 | 0.14 |
| OA | Hippocampus | | Amygdala | | S1 | |
|  | Left | Right | Left | Right | Left | Right |
| pFear (r) | 0.43 | 0.42 | 0.25 | 0.20 | 0.24 | 0.20 |
| p-value | 0.06 | 0.07 | 0.29 | 0.40 | 0.31 | 0.40 |
| eFear (r) | 0.26 | 0.26 | 0.19 | 0.24 | 0.49 | 0.33 |
| p-value | 0.27 | 0.27 | 0.43 | 0.31 | 0.03 | 0.16 |
